# Supplementary material for: Ventral pallidal GABAergic neurons control hedonic feeding and obesity
Source: bioRxiv. 2026 Jun 23:2026.06.18.733195. Preprint. [Version 1] doi: 10.64898/2026.06.18.733195 (PMC13320888; doi:10.64898/2026.06.18.733195)
Supplement: Supplement 3 — Supplemental Table 1. Full statistics and animal numbers for Figure 1–4 and S1-10. [file media-3.pdf]

Supplemental Table 1.

Figure	Measure	Test	Comparison	n (M/F)	df	Test statistic	p-value	Post-hoc p-values	Effect size
**Figure 1. Arc^{AgRP} and VP^{GABA} neurons activate distinct feeding drives.**									
1B	Arc^{AgRP} chow consumption	One-way RM-ANOVA + Bonferroni post-hoc	PRE vs STIM vs POST	6 (3M/3F)	F(2,10)	F = 65.20	1.8e-06	PRE-STIM 6.1e-4<br>STIM-POST 8.8e-4<br>PRE-POST 0.102	η^{2}p = 0.929; STIM-vs-PRE d_z = -3.94
1C	Arc^{AgRP} HFD consumption	One-way RM-ANOVA + Bonferroni post-hoc	PRE vs STIM vs POST	6 (3M/3F)	F(2,10)	F = 35.69	2.8e-05	PRE-STIM 3.6e-3<br>STIM-POST 0.013<br>PRE-POST 3.9e-3	η^{2}p = 0.877; STIM-vs-PRE d_z = -2.70
1D	Arc^{AgRP} chow prestimulation	Paired t-test	laser OFF vs ON (STIM) (pre-access)	6 (3M/3F)	t(5)	t = -6.442	1.3e-03	—	Cohen's d_z = -2.63
1D	Arc^{AgRP} HFD prestimulation	Paired t-test	laser OFF vs ON (STIM) (pre-access)	6 (3M/3F)	t(5)	t = -5.317	3.2e-03	—	Cohen's d_z = -2.17
1F	VP^{GABA} chow consumption	One-way RM-ANOVA + Bonferroni post-hoc	PRE vs STIM vs POST	12 (5M/7F)	F(2,22)	F = 5.595	0.011	PRE-STIM 0.093<br>STIM-POST 0.135<br>PRE-POST 0.288	η^{2}p = 0.337; STIM-vs-PRE d_z = -0.72
1F	VP^{GABA} chow consumption — Males [sex-split]	One-way RM-ANOVA + Bonferroni post-hoc	PRE vs STIM vs POST	5 (5M/0F)	F(2,8)	F = 0.961	0.423 (ns)	ON-vs-PRE 0.981	η^{2}p = 0.194; STIM-vs-PRE d_z = -0.50
1F	VP^{GABA} chow consumption — Females [sex-split]	One-way RM-ANOVA + Bonferroni post-hoc	PRE vs STIM vs POST	7 (0M/7F)	F(2,12)	F = 4.805	0.029	ON-vs-PRE 0.213	η^{2}p = 0.445; STIM-vs-PRE d_z = -0.83
1G	VP^{GABA} HFD consumption	One-way RM-ANOVA + Bonferroni post-hoc	PRE vs STIM vs POST	12 (5M/7F)	F(2,22)	F = 31.23	3.8e-07	PRE-STIM 5.1e-4<br>STIM-POST 3.9e-4<br>PRE-POST 0.358	η^{2}p = 0.740; STIM-vs-PRE d_z = -1.61
1G	VP^{GABA} HFD consumption — Males [sex-split]	One-way RM-ANOVA + Bonferroni post-hoc	PRE vs STIM vs POST	5 (5M/0F)	F(2,8)	F = 8.491	0.011	STIM-vs-PRE 0.110	η^{2}p = 0.680; STIM-vs-PRE d_z = -1.38
1G	VP^{GABA} HFD consumption — Females [sex-split]	One-way RM-ANOVA + Bonferroni post-hoc	PRE vs STIM vs POST	7 (0M/7F)	F(2,12)	F = 21.59	1.1e-04	STIM-vs-PRE 0.014	η^{2}p = 0.783; STIM-vs-PRE d_z = -1.66
1H	VP^{GABA} chow prestimulation	Paired t-test	laser OFF vs ON (STIM) (pre-access)	8 (4M/4F)	t(7)	t = -0.321	0.758 (ns)	—	Cohen's d_z = -0.11
1H	VP^{GABA} HFD prestimulation	Paired t-test	laser OFF vs ON (STIM) (pre-access)	8 (4M/4F)	t(7)	t = 0.351	0.736 (ns)	—	Cohen's d_z = 0.12
1**L**	Arc^{AgRP} photometry AUC	One-way RM-ANOVA (parametric; Shapiro W=0.942, p=0.133)	saline vs ghrelin vs CCK	9 (5M/4F)	F(2,16)	F = 56.56	5.6e-08	(see post-hoc rows below)	η^{2}p = 0.876
1**L**	Arc^{AgRP} ghrelin vs saline-baseline	Paired t-test (Holm-corrected)	ghrelin vs saline	9 (5M/4F)	t(8)	raw p = 2.9e-4	adj p = 5.8e-4	—	Cohen's d_z = -2.03
1**L**	Arc^{AgRP} CCK vs saline-baseline	Paired t-test (Holm-corrected)	CCK vs saline	9 (5M/4F)	t(8)	raw p = 4.5e-4	adj p = 5.8e-4	—	Cohen's d_z = 1.90
1**L**	Arc^{AgRP} CCK vs ghrelin	Paired t-test (Holm-corrected)	CCK vs ghrelin	9 (5M/4F)	t(8)	raw p = 1.7e-5	adj p = 5.2e-5	—	Cohen's d_z = 3.03
1**O**	VP^{GABA} photometry AUC	One-way RM-ANOVA (parametric; Shapiro W=0.965, p=0.401)	saline vs ghrelin vs CCK	10 (3M/7F)	F(2,18)	F = 12.58	3.8e-04	(see post-hoc rows below)	η^{2}p = 0.583
1**O**	VP^{GABA} ghrelin vs saline-baseline	Paired t-test (Holm-corrected)	ghrelin vs saline	10 (3M/7F)	t(9)	raw p = 0.124	adj p = 0.124 (ns)	—	Cohen's d_z = 0.54
1**O**	VP^{GABA} CCK vs saline-baseline	Paired t-test (Holm-corrected)	CCK vs saline	10 (3M/7F)	t(9)	raw p = 1.2e-3	adj p = 3.6e-3	—	Cohen's d_z = 1.47
1**O**	VP^{GABA} CCK vs ghrelin	Paired t-test (Holm-corrected)	CCK vs ghrelin	10 (3M/7F)	t(9)	raw p = 5.8e-3	adj p = 0.012	—	Cohen's d_z = 1.14
**Figure 2. VP^{GABA} neurons bi-directionally control reward consumption.**									
**2C**	Licks per block	Paired t-test	multi-stim activation, laser OFF vs ON	8 (4M/4F)	t(7)	t = 3.149	0.0162	—	Cohen's d_z = 1.11
**2C**	Bouts per block	Paired t-test	multi-stim activation, laser OFF vs ON	8 (4M/4F)	t(7)	t = 6.581	3e-4	—	Cohen's d_z = 2.33
**2C**	Average bout duration	Paired t-test	multi-stim activation, laser OFF vs ON	8 (4M/4F)	t(7)	t = 3.086	0.0177	—	Cohen's d_z = 1.09
**2D**	Licks per block	Paired t-test	continuous activation, laser OFF vs ON	11 (7M/4F)	t(10)	t = 3.996	2.5e-3	—	Cohen's d_z = 1.21
**2D**	Bouts per block	Paired t-test	continuous activation, laser OFF vs ON	11 (7M/4F)	t(10)	t = 4.138	2e-3	—	Cohen's d_z = 1.25
**2D**	Average bout duration	Paired t-test	continuous activation, laser	11 (7M/4F)	t(10)	t = 2.741	0.0208	—	Cohen's d_z = 0.83

			OFF vs ON						
2**E**	Licks per block	Paired t-test	constant inhibition (GtACR2), laser OFF vs ON	9 (6M/3F)	t(8)	t = -3.184	0.0129	—	Cohen's d_z = -1.061
2**E**	Bouts per block	Paired t-test	constant inhibition (GtACR2), laser OFF vs ON	9 (6M/3F)	t(8)	t = -2.902	0.0198	—	Cohen's d_z = -0.967
2**E**	Average bout duration	Paired t-test	constant inhibition (GtACR2), laser OFF vs ON	9 (6M/3F)	t(8)	t = -3.401	9.3e-3	—	Cohen's d_z = -1.134
2**F**-**H**	$VP^{GABA:ChR2}$ single-unit classification	One-sample Wilcoxon vs 0 per unit, BH-FDR (α=0.10), |median ΔFR|>1 Hz gate	post-stim ΔFR vs 0, per untagged single unit	10 untagged SU (n=3 mice, 3M/0F)	per-unit (40 bins)	W = 18–387	per-unit p_FDR 1.7e-5–0.757	BH-FDR	0 activated / 5 inhibited / 5 unmodulated; + 3 optically tagged units (defined upstream by light-evoked latency) → 13 single units total
**Figure 3. Single-photon endoscopic imaging reveals functionally heterogeneous VP^{GABA} dynamics that encode and track consummatory licking.**									
3C	Combined cell classification (lick-bouts 0–3 s)	One-sample Wilcoxon vs 0 per cell, BH-FDR	post-onset z-score vs 0 (339 cells)	8 (7M/1F); 339 cells	per-cell	median raw p = 5.6e-09	297/339 sig (FDR)	—	129 activated / 168 inhibited / 42 unmodulated
3E	Cell classification, single licks (0–3 s)	One-sample Wilcoxon vs 0 per cell, BH-FDR	post-event z-score vs 0 (339 cells)	8 (7M/1F); 339 cells	per-cell	median raw p = 5.6e-05	252/339 sig (FDR)	—	110 activated / 142 inhibited / 87 unmodulated
3E	Cell classification, short bouts (0–6 s)	One-sample Wilcoxon vs 0 per cell, BH-FDR	post-event z-score vs 0 (339 cells)	8 (7M/1F); 339 cells	per-cell	median raw p = 9.0e-08	294/339 sig (FDR)	—	141 activated / 153 inhibited / 45 unmodulated
3E	Cell classification, long bouts (0–15 s)	One-sample Wilcoxon vs 0 per cell, BH-FDR	post-event z-score vs 0 (339 cells)	8 (7M/1F); 339 cells	per-cell	median raw p = 1.3e-25	323/339 sig (FDR)	—	151 activated / 172 inhibited / 16 unmodulated
3F	Cell response composition	Chi-square test of independence	act/unmod/inh × single/short/long	8 (7M/1F); 339 cells	$\chi^2(4)$	χ^2 = 61.44	1.4e-12	—	Cramér's V = 0.174
3F	Mean activated amplitude	One-way RM-ANOVA (per-mouse) + Holm paired t	single vs short vs long bout	8 (7M/1F)	F(2,14)	F = 25.34	2.2e-5	S-Sh 0.040; S-L 0.0028; Sh-L 0.0038; s – single, Sh – short, L – long	η^2p = 0.784
3F	Mean inhibited amplitude	One-way RM-ANOVA (per-mouse) + Holm paired t	single vs short vs long bout	8 (7M/1F)	F(2,14)	F = 11.07	1.3e-3	S-Sh 0.313; S-L 0.031; Sh-L 0.023	η^2p = 0.613
3H	Licks per block	Paired t-test	closed-loop, laser OFF vs ON	15 (9M/6F)	t(14)	t = 3.659	2.6e-3	—	Cohen's d_z = 0.945
3**I**	Bouts per block	Paired t-test	closed-loop, laser OFF vs ON	15 (9M/6F)	t(14)	t = -1.674	0.1164 (ns)	—	Cohen's d_z = 0.432
3**J**	Average bout duration	Paired t-test	closed-loop, laser OFF vs ON	15 (9M/6F)	t(14)	t = 6.632	<0.0001	—	Cohen's d_z = 1.712
**Figure 4. VP^{GABA} neurons are required for the development of diet-induced obesity.**									
4B	slc17a6 (VGLUT2) transcript count	Independent t-test	mCherry vs taCasp3	mCh 5 (2M/3F); casp 5 (2M/3F)	t(8)	t = -0.321	0.757 (ns)	—	Cohen's d = -0.20
4B	slc32a1 (VGAT/GABA) transcript count	Independent t-test	mCherry vs taCasp3	mCh 5 (2M/3F); casp 5 (2M/3F)	t(8)	t = 4.721	1.5e-03	—	Cohen's d = 2.99
4C	Cumulative licks at 15 min‡	Independent t-test	mCherry vs taCasp3	mCh 11 (5M/6F); casp 13 (8M/5F)	t(22)	t = 2.885	8.6e-03	—	Cohen's d = 1.18
4E	Avg lick bouts/session	Independent t-test	mCherry vs taCasp3	mCh 11 (5M/6F); casp 13 (8M/5F)	t(22)	t = 2.636	0.015	—	Cohen's d = 1.08
4E	Mean lick-bout duration	Independent t-test	mCherry vs taCasp3	mCh 11 (5M/6F); casp 13 (8M/5F)	t(22)	t = 0.870	0.393 (ns)	—	Cohen's d = 0.36
4F	Per-mouse modal lick frequency	Independent t-test	mCherry vs taCasp3	mCh 11 (5M/6F); casp 13 (8M/5F)	t(22)	t = 3.091	5.3e-03	—	Cohen's d = 1.27
4G	Diet consumption Group effect	Mixed RM-ANOVA (between Group, within State)	mCherry vs taCasp3	18 (10 mCh / 8 casp)	F(1,16)	F = 1.789	0.200 (ns)	—	η^2p = 0.101
4G	Diet consumption State effect	Mixed RM-ANOVA	Sated vs Fasted	18	F(1,16)	F = 59.53	8.8e-07	—	η^2p = 0.788
4G	Diet consumption Group × State	Mixed RM-ANOVA	Group × State interaction	18	F(1,16)	F = 6.694	0.020	—	η^2p = 0.295
4G	Consumption post-hoc (Sated)	Mann-Whitney U, Holm-corrected	mCherry vs taCasp3 (Sated)	mCh 8 / casp 10	—	U = 67.0	raw 0.0185	p_Holm = 0.037	rank-biserial r = -0.68
4G	Consumption post-hoc (Fasted)	Mann-Whitney U, Holm-corrected	mCherry vs taCasp3 (Fasted)	mCh 8 / casp 10	—	U = 41.5	raw 0.929	p_Holm = 0.929 (ns)	rank-biserial r = -0.04
4I	HFD weight Wk1→Wk5, mCherry-HFD	Paired t-test	week 1 vs week 5	23 (15M/8F)	t(22)	t = -4.538	1.6e-04	—	Cohen's d_z = -0.95
4I	HFD weight Wk1→Wk5, taCasp3-HFD	Paired t-test	week 1 vs week 5	22 (17M/5F)	t(21)	t = 1.269	0.218 (ns)	—	Cohen's d_z = 0.27
4I	HFD weight Wk1→Wk5, taCasp3-chow	Paired t-test	week 1 vs week 5	7 (2M/5F)	t(6)	t = -1.747	0.131 (ns)	—	Cohen's d_z = -0.66
4J	HFD weight Wk5→Wk10, mCherry-HFD	Paired t-test	week 5 vs week 10	23 (15M/8F)	t(22)	t = -8.752	1.1e-08	—	Cohen's d_z = -1.83
4J	HFD weight Wk5→Wk10, taCasp3-HFD	Paired t-test	week 5 vs week 10	22 (17M/5F)	t(21)	t = -6.531	1.7e-06	—	Cohen's d_z = -1.39
4J	HFD weight Wk5→Wk10, taCasp3-chow	Paired t-test	week 5 vs week 10	7 (2M/5F)	t(6)	t = -2.969	0.025	—	Cohen's d_z = -1.12
4J	HFD weight Wk10 group comparison	Mann-Whitney U	mCherry-HFD vs taCasp3-HFD at week 10	mCh-HFD 23 (15M/8F) / casp-HFD 22 (17M/5F)	—	U = 427.0	8.2e-05	—	rank-biserial r = -0.69
**4J**	HFD weight Wk10 group	Mann-Whitney U	mCherry-HFD vs taCasp3-	mCh-HFD (15M) /	—	U = 221.0	<0.001	—	rank-biserial r = -0.733

	comparison (sex split, males)		HFD at week 10	casp-HFD (17M)					
**4J**	HFD weight Wk10 group comparison (sex split, females)	Mann-Whitney U	mCherry-HFD vs taCasp3-HFD at week 10	mCh-HFD (8F) / casp-HFD (5F)	—	U = 39.0	0.003	—	rank-biserial r = -0.950
**Supplemental Figure 1. Behavioral ethograms and locomotor tracking during optogenetic VP^{GABA} stimulation.**									
S1B	Chow interaction duration	One-way RM-ANOVA + Holm post-hoc	PRE vs STIM vs POST	10 (6M/4F)	F(2,18)	F = 6.254	0.0087	PRE-STIM 0.095<br>STIM-POST 0.095<br>PRE-POST 0.505	η^2p = 0.410; STIM-vs-PRE d_z = -0.80
S1B	HFD interaction duration	One-way RM-ANOVA + Holm post-hoc	PRE vs STIM vs POST	10 (6M/4F)	F(2,18)	F = 9.199	0.0018	PRE-STIM 0.042<br>STIM-POST 0.042<br>PRE-POST 0.933	η^2p = 0.506; STIM-vs-PRE d_z = -0.96
S1B	Non-food object interaction duration	One-way RM-ANOVA + Holm post-hoc	PRE vs STIM vs POST	9 (5M/4F)	F(2,16)	F = 4.046	0.0378	PRE-ON 0.231<br>ON-POST 0.231<br>PRE-POST 0.924	η^2p = 0.336; ON-vs-PRE d_z = -0.67
S1D	Average locomotor speed	Paired t-test	laser OFF vs ON	8 (4M/4F)	t(7)	t = -0.872	0.412 (ns)	—	Cohen's d_z = -0.31
S1D	Time in arena centre	Paired t-test	laser OFF vs ON	8 (4M/4F)	t(7)	t = -2.462	0.043	—	Cohen's d_z = -0.87
**Supplemental Figure 2. Single cell RNA-sequencing of the VP and whole-brain mapping of fasting-activated inputs to the VP.**									
S2A	Feeding gene enrichment between Arc and VP	AddModuleScore (Seurat); n=100 control gene randomly selected	Arc vs. VP expression	—	—	—	—	—	—
S2D-F	Whole brain cFos counts	Light sheet imaging + cFos nuclei counting	See table S2	8 (8M/0F), per group	See table S2	See table S2	See table S2	See table S2	See table S2
**Supplemental Figure 3. Fiber photometry of Arc^{AgRP} and VP^{GABA} neurons aligned to pellet retrievals dispensed from FED3.**									
S3B	Post-injection pellet intake	Paired t-test	Saline vs ghrelin	9 (7M/2F)	t(8)	t = 12.48	1.6e-6	—	Cohen's d_z = 4.16
S3E	Peak deflection (-1s to +1s)	Welch's t-test	Arc^{AgRP} vs VP^{GABA} (between-subjects)	Arc^{AgRP} (9, 5M/4F); VP^{GABA} (6, 4M/2F)	df = 11.43	t = -18.394	<0.0001	—	d = -8.982
S3F	Post-event AUC (Arc^{AgRP})	Paired t-test	First vs last retrieval	9 (5M/4F)	t(8)	t = -3.851	4.9e-03	—	Cohen's d_z = -1.284
S3G	Post-event AUC (VP^{GABA})	Paired t-test	First vs last retrieval	6 (4M/2F)	t(5)	t = 1.837	0.1257	—	Cohen's d_z = 0.750
**Supplemental Figure 4. VP^{GABA} optical activation and inhibition drive preference and avoidance, respectively.**									
S4C	Time on stim-paired side (%), ChR2	Paired t-test	baseline (OFF) vs laser-ON	13 (8M/5F)	t(12)	t = -7.068	1.3e-05	—	Cohen's d_z = -1.96
S4F	Time on stim-paired side (%), GtACR2	Paired t-test	baseline (OFF) vs laser-ON	8 (5M/3F)	t(7)	t = 3.974	5.4e-03	—	Cohen's d_z = 1.41
**Supplemental Figure 5. Optogenetic activation of VP^{GABA} neurons drives licking for quinine and an empty spout.**									
S5B	Quinine licks per block	Paired t-test	laser OFF vs ON	8 (4M/4F)	t(7)	t = 5.761	6.9e-04	—	Cohen's d_z = 2.04
S5B	Quinine bouts per block	Paired t-test	laser OFF vs ON	8 (4M/4F)	t(7)	t = 4.732	2.1e-03	—	Cohen's d_z = 1.67
S5B	Quinine average bout duration	Paired t-test	laser OFF vs ON	8 (4M/4F)	t(7)	t = 5.008	1.6e-03	—	Cohen's d_z = 1.77
S5D	Empty licks per block	Paired t-test	laser OFF vs ON	10 (5M/5F)	t(9)	t = 3.324	8.9e-03	—	Cohen's d_z = 1.05
S5D	Empty bouts per block	Paired t-test	laser OFF vs ON	10 (5M/5F)	t(9)	t = 4.267	2.1e-03	—	Cohen's d_z = 1.35
S5D	Empty average bout duration	Paired t-test	laser OFF vs ON	10 (5M/5F)	t(9)	t = 3.329	8.8e-03	—	Cohen's d_z = 1.05
S5F	Cross-condition licks per block	One-way ANOVA + Welch post-hoc (uncorrected)	quinine vs empty vs boost (laser-ON)	29 (16M/13F)	F(2,26)	F = 4.855	0.016	q-empty 0.138<br>q-boost 0.0077<br>empty-boost 0.0775	η^2p = 0.272
S5F	Cross-condition bouts per block	One-way ANOVA	quinine vs empty vs boost	29 (16M/13F)	F(2,26)	F = 0.494	0.616 (ns)	—	η^2p = 0.037
S5F	Cross-condition average bout duration	One-way ANOVA + Welch post-hoc (uncorrected)	quinine vs empty vs boost	29 (16M/13F)	F(2,26)	F = 2.984	0.068 (ns)	q-empty 0.071; q-boost 0.039; empty-boost 0.164	η^2p = 0.187
S5G	Cross-condition modal lick freq	One-way ANOVA	quinine vs empty vs boost	29 (16M/13F)	F(2,26)	F = 0.981	0.388 (ns)	—	η^2p = 0.070
**Supplemental Figure 6. Principal component state dynamics of VP^{GABA} neurons and linear decoding of VP^{GABA} population dynamics during self-paced drinking.**									
S6C	Coefficients of PC1 eigenvector	Top and bottom quartile (25% hi/lo)	—	—	—	—	—	—	—
S6D	High PC1 (top 25%) post-event AUC	Paired t-test (Holm)	Single vs short	84 cells (8 mice, 7M/1F)	t(83)	t = -6.006	< 0.0001	< 0.0001	Cohen's d_z = -0.655
S6D	High PC1 (top 25%) post-event AUC	Paired t-test (Holm)	Single vs long	84 cells (8 mice, 7M/1F)	t(83)	t = -8.100	< 0.0001	< 0.0001	Cohen's d_z = -0.884
S6D	High PC1 (top 25%) post-event AUC	Paired t-test (Holm)	Short vs long	84 cells (8 mice, 7M/1F)	t(83)	t = -5.596	< 0.0001	< 0.0001	Cohen's d_z = -0.611
S6D	Low PC1 (bottom 25%) post-event AUC	Paired t-test (Holm)	Single vs short	84 cells (8 mice, 7M/1F)	t(83)	t = 7.943	< 0.0001	< 0.0001	Cohen's d_z = 0.867
S6D	Low PC1 (bottom 25%) post-event AUC	Paired t-test (Holm)	Single vs long	84 cells (8 mice, 7M/1F)	t(83)	t = 17.027	< 0.0001	< 0.0001	Cohen's d_z = 1.858
S6D	Low PC1 (bottom 25%) post-event AUC	Paired t-test (Holm)	Short vs long	84 cells (8 mice, 7M/1F)	t(83)	t = 13.370	< 0.0001	< 0.0001	Cohen's d_z = 1.459
S6G, H	Balanced accuracy vs shuffle (time resolved)	Wilcoxon signed rank per dt (BH-FDR)	Decoder > shuffle	8 (7M/1F); 13 sessions	—	—	—	Min FDR p = 0.0004	—
S6I	auROC, -3 to +0.5 s window	One-sample Wilcoxon vs 0.5 (BH-FDR)	per-session auROC vs chance	8 (7M/1F); 13 sessions	n=13	median auROC = 0.490	p_raw 0.787; FDR 0.893 (ns)	—	median 0.490 vs 0.5
S6I	auROC, -3 to +1 s window	One-sample Wilcoxon vs 0.5	per-session auROC vs	8 (7M/1F); 13	n=13	median auROC	p_raw 0.893;	—	median 0.490 vs 0.5

		(BH-FDR)	chance	sessions		= 0.490	FDR 0.893 (ns)		
S6I	auROC, -3 to +3 s window	One-sample Wilcoxon vs 0.5 (BH-FDR)	per-session auROC vs chance	8 (7M/1F); 13 sessions	n=13	median auROC = 0.595	p_raw 0.216; FDR 0.433 (ns)	—	median 0.595 vs 0.5
S6I	auROC, -3 to +10 s window	One-sample Wilcoxon vs 0.5 (BH-FDR)	per-session auROC vs chance	8 (7M/1F); 13 sessions	n=13	median auROC = 0.731	p_raw 1.2e-3; FDR 4.9e-3	—	median 0.731 vs 0.5
**Supplemental Figure 7. VP^{GABA} ablation leaves homeostatic weight regulation intact, but HFD exposure in VP^{GABA} ablated mice induces hyperglycemia.**									
S7B	Chow weight Wk1→Wk5, mCherry-chow	Paired t-test	week 1 vs week 5	5 (0M/5F)	t(4)	t = -0.787	0.475 (ns)	—	Cohen's d_z = -0.35
S7B	Chow weight Wk1→Wk5, taCasp3-chow	Paired t-test	week 1 vs week 5	7 (2M/5F)	t(6)	t = -1.747	0.131 (ns)	—	Cohen's d_z = -0.66
S7C	Chow weight Wk5→Wk10, mCherry-chow	Paired t-test	week 5 vs week 10	5 (0M/5F)	t(4)	t = -3.633	0.022	—	Cohen's d_z = -1.63
S7C	Chow weight Wk5→Wk10, taCasp3-chow	Paired t-test	week 5 vs week 10	7 (2M/5F)	t(6)	t = -2.969	0.025	—	Cohen's d_z = -1.12
S7C	Chow weight Wk10 group comparison	Mann-Whitney U	control-chow vs taCasp3-chow at week 10	ctrl-chow 5 / casp-chow 7	—	U = 27.0	0.149 (ns)	—	rank-biserial r = -0.54
S7F	Average daily pellets	Independent t-test (two-tailed)	mCherry vs taCasp3	mCh 5 (0M/5F); casp 7 (2M/5F)	t(10)	t = -1.184	0.264 (2-tailed); 0.868 (1-tailed)	—	Cohen's d = -0.69
S7H	GTT area under the curve	One-way ANOVA + Tukey HSD	chow (mCherry, taCasp3 combined) vs mCherry-HFD vs taCasp3-HFD	43: chow 12 (2M/10F); mCh-HFD 14 (8M/6F); casp-HFD 17 (10M/7F)	F(2,40)	F = 30.94	7.6e-09	chow vs mCh-HFD <0.001<br>chow vs casp-HFD <0.001<br>mCh-HFD vs casp-HFD 0.0173	η²p = 0.607
**Supplemental Figure 8. VP^{GABA} ablation does not disrupt circadian feeding patterns.**									
S8A	Cumulative hopper touches (72 h)	Independent t-test (two-tailed)	mCherry vs taCasp3	31 (18M/13F): mCh 16 (8/8); casp 15 (10/5)	t(29)	t = -0.498	0.622 (ns)	—	Cohen's d = -0.18
S8D	Daily kcal consumed	Independent t-test (one-tailed)	control vs taCasp3	42 (31M/11F): ctrl 21 (14/7); casp 21 (17/4)	t(40)	t = 1.881	0.034 (1-tailed); 0.067 (2-tailed)	—	Cohen's d = 0.58
S8E	Daily meal counts	Independent t-test (one-tailed)	mCherry vs taCasp3	31 (18M/13F): mCh 16; casp 15	t(29)	t = -0.389	0.700 (2-tailed)	—	Cohen's d = -0.14
S8F	Average meal duration	Independent t-test (one-tailed)	mCherry vs taCasp3	31 (18M/13F): mCh 16; casp 15	t(29)	t = 0.817	0.420 (2-tailed)	—	Cohen's d = 0.29
**Supplemental Figure 9. Effect of VP^{GABA} ablation on operant reward seeking and reversal learning.**									
S9C	Pokes per pellet (animal-averaged)	Independent t-test	taCasp3 vs mCherry	12 (taCasp3 7 / mCh 5)	t(10)	t = 0.538	0.602 (ns)	—	Cohen's d = 0.32
S9C	Median break point (animal-averaged)	Independent t-test	taCasp3 vs mCherry	12 (taCasp3 7 / mCh 5)	t(10)	t = 0.610	0.556 (ns)	—	Cohen's d = 0.36
S9G	Pre-reversal accuracy	Independent t-test	mCherry vs taCasp3	12 (5/7)	t(10)	t = 2.278	0.046	—	Cohen's d = 1.33
S9H	Pellets/day	Independent t-test (two-sided)	mCherry vs taCasp3	12 (5/7)	t(10)	t = 0.097	0.924 (ns)	—	Cohen's d = 0.06
S9I	Logistic-regression OR, lag t-5	Independent t-test	mCherry vs taCasp3	12 (5/7)	t(10)	t = 0.760	0.465 (ns)	—	Cohen's d = 0.45
S9I	Logistic-regression OR, lag t-4	Independent t-test	mCherry vs taCasp3	12 (5/7)	t(10)	t = 1.746	0.111 (ns)	—	Cohen's d = 1.02
S9I	Logistic-regression OR, lag t-3	Independent t-test	mCherry vs taCasp3	12 (5/7)	t(10)	t = -0.605	0.559 (ns)	—	Cohen's d = -0.35
S9I	Logistic-regression OR, lag t-2	Independent t-test	mCherry vs taCasp3	12 (5/7)	t(10)	t = 0.995	0.343 (ns)	—	Cohen's d = 0.58
S9I	Logistic-regression OR, lag t-1	Independent t-test	mCherry vs taCasp3	12 (5/7)	t(10)	t = 1.844	0.095 (ns)	—	Cohen's d = 1.08
S9J	Win-stay	Independent t-test	mCherry vs taCasp3	12 (5/7)	t(10)	t = 1.846	0.095 (ns)	—	Cohen's d = 1.08
S9K	Lose-stay	Independent t-test	mCherry vs taCasp3	12 (5/7)	t(10)	t = 0.668	0.520 (ns)	—	Cohen's d = 0.39
**Supplemental Figure 10. Social seeking behaviors remain intact following VP^{GABA} ablation.**									
S10B	Average social seeking event (Day 2-4 averaged per animal)	Mann-Whitney U	mCherry vs. taCasp3	5 mCh (0M/5F); 7 casp (2M/5F)	—	U = 20	0.755 (ns)	—	Rank-biserial r = 0.143
S10D	Social seeking AUC during the dark cycle (Day 2-4 averaged per animal)	Mann-Whitney U	mCherry vs. taCasp3	5 mCh (0M/5F); 7 casp (2M/5F)	—	U = 22	0.530 (ns)	—	Rank-biserial r = -0.257
S10F	Percentage of approach trials after nose poke (Day 2-4)	Mann-Whitney U	mCherry vs. taCasp3	5 mCh (0M/5F); 7 casp (2M/5F)	—	U = 15	0.755 (ns)	—	Rank-biserial r = -0.143
S10G	Median approach latency after nose poke (Day 2-4)	Mann-Whitney U	mCherry vs. taCasp3	5 mCh (0M/5F); 7 casp (2M/5F)	—	U = 30	0.048	—	Rank-biserial r – 0.714
S10D	Home-cage activity, lights OFF (Night)	Mann-Whitney U, Holm-corrected	mCherry vs taCasp3	45 (32M/13F): mCh 23; casp 22	—	U = 116.0	raw 0.0019; Holm 0.0039	—	rank-biserial r = 0.54; d = -1.11
